# Mechanical Strain Causes Adaptive Change in Bronchial Fibroblasts Enhancing Profibrotic and Inflammatory Responses

**DOI:** 10.1371/journal.pone.0153926

**Published:** 2016-04-21

**Authors:** Wiparat Manuyakorn, David E. Smart, Antonio Noto, Fabio Bucchieri, Hans Michael Haitchi, Stephen T. Holgate, Peter H. Howarth, Donna E. Davies

**Affiliations:** 1 Clinical and Experimental Sciences, Sir Henry Wellcome Laboratories, University of Southampton School of Medicine, Southampton General Hospital, Southampton, United Kingdom; 2 National Institute for Health Research (NIHR) Southampton Respiratory Biomedical Research Unit, Southampton Centre for Biomedical Research, Southampton General Hospital, Southampton, United Kingdom; 3 BIONEC Department, University of Palermo, Palermo, Italy; 4 Istituto Euro-Mediterraneo di Scienza e Tecnologia, IEMEST, Palermo, Italy; Medical University of South Carolina, UNITED STATES

## Abstract

Asthma is characterized by periodic episodes of bronchoconstriction and reversible airway obstruction; these symptoms are attributable to a number of factors including increased mass and reactivity of bronchial smooth muscle and extracellular matrix (ECM) in asthmatic airways. Literature has suggested changes in cell responses and signaling can be elicited via modulation of mechanical stress acting upon them, potentially affecting the microenvironment of the cell. In this study, we hypothesized that mechanical strain directly affects the (myo)fibroblast phenotype in asthma. Therefore, we characterized responses of bronchial fibroblasts, from 6 normal and 11 asthmatic non-smoking volunteers, exposed to cyclical mechanical strain using flexible silastic membranes. Samples were analyzed for proteoglycans, α-smooth muscle actin (αSMA), collagens I and III, matrix metalloproteinase (MMP) 2 & 9 and interleukin-8 (IL-8) by qRT-PCR, Western blot, zymography and ELISA. Mechanical strain caused a decrease in αSMA mRNA but no change in either αSMA protein or proteoglycan expression. In contrast the inflammatory mediator IL-8, MMPs and interstitial collagens were increased at both the transcriptional and protein level. The results demonstrate an adaptive response of bronchial fibroblasts to mechanical strain, irrespective of donor. The adaptation involves cytoskeletal rearrangement, matrix remodelling and inflammatory cytokine release. These results suggest that mechanical strain could contribute to disease progression in asthma by promoting inflammation and remodelling responses.

## Introduction

Asthma is a common chronic disorder of the conducting airways that is characterized by bronchial hyper-responsiveness (BHR) and reversible airflow obstruction in association with underlying airway inflammation and remodelling. Inflammation of the airways is typically associated with an influx of eosinophils and accompanied by elevation of Th2 cytokines [1]. In chronic asthma, the airways are also remodelled due to an increase in smooth muscle mass, deposition of extracellular matrix (ECM) proteins including collagens and proteoglycans, and neoangiogenesis accompanied by micro-vascular leakage [2]. Although inflammation has been viewed as a key driver for airway remodelling and BHR, there is a poor correlation between inflammation, damage, functional impairment and degree of symptoms [3]. In a recent *in vivo* challenge study, it has been demonstrated that methacholine-induced bronchoconstriction is sufficient to increase sub-epithelial collagen in asthmatic airways in the absence of increased airway inflammation, suggesting that mechanical forces alone can contribute significantly to matrix remodelling responses seen in asthma. Moreover, the data imply that remodelling may be a therapeutic target in its own right [4, 5].

The ECM has important roles in determining the mechanical properties and elasticity of a tissue. Since the ECM compartment is dynamic, reflecting the net balance of synthesis and degradation, a shift in this balance towards increased matrix deposition results in ‘fibrosis’ leading to altered structure and abnormal mechanical properties [6]. Fibroblasts are one of the major cell types responsible for transferring mechanical signals into biological events, especially expression of ECM genes [7]. Abnormal mechanical loads can affect diverse cellular functions including cell proliferation and alteration of the composition of the ECM leading to fibrosis [7]. Previous studies have highlighted the role of airway fibroblasts in ECM production in response to mechanical stress [8–10]. Although BHR has been shown to be inversely related to the airway wall thickness [11], as yet there are few studies characterizing disease-related differences in the responses of airway fibroblasts from non-asthmatic or asthmatic subjects to mechanical strain.

In view of the epithelial sensitivity to mechanical strain in asthma [4], we postulated that airway fibroblasts from asthmatic subjects would have a modified response to mechanical strain when compared with fibroblasts from non-asthmatic subjects. To test our hypothesis, we studied the effect of mechanical strain on expression of ECM components and proinflammatory cytokines by primary bronchial fibroblasts obtained from 11 asthmatic and 6 non-asthmatic non-smoking subjects. We measured changes in collagens I and III, versican, and decorin as markers of ECM expression, MMP-2 and MMP-9 as markers of matrix turnover and IL-8 as a marker of a proinflammatory response. Since mechanical strain has been reported to affect smooth muscle differentiation [12], we also investigated whether mechanical strain could promote fibroblast/myofibroblast differentiation by measuring αSMA expression.

## Methods

### Bronchial fibroblasts

Primary fibroblasts were obtained from bronchial biopsies harvested in line with methodology consistent with nationally established guidelines [13] following local Institutional (No: 123/01) and regional ethical approval by the Southampton and South West Research Ethics Committee (REC No. 05/Q1702/165). All donor samples were obtained following clinician informed written consent and were anonymised with a donor code in line with aforementioned ethical approval for research. Primary fibroblasts from asthmatic and normal volunteers were produced as outgrowths from the bronchial biopsies [14]. The clinical characterization of the two subject groups showed them to have both similar mean lung function and mean age. The lung functions FEV_1_% predicted (±SE) for the non-asthmatic (n = 6) and asthmatics (n = 11) were 103.7±6.3 and 87.7±10.0 respectively with the mean ages for the two groups being 37±19.9 and 39.8±17.6 years. To obtain fibroblast outgrowths, biopsies were placed in a petri dish with 10% FBS/Dulbecco's modified Eagle's medium (DMEM) containing 50 IU/ml penicillin, 50 μg/ml streptomycin, 2 mM L-glutamine, 1mM sodium pyruvate and 1mM non-essential amino acids and were chopped into small pieces using sterile scalpel blades; this process also scored the bottom of the dish which provided anchoring points for the tissue fragments. The tissue fragments were incubated in a humidified incubator at 37°C, 5% CO_2_, for approximately 1 week, during which time fibroblasts migrated from the tissue and proliferated on the base of the culture dish. The fibroblast cultures were then passaged weekly using 1% trypsin and experiments were performed using cells between passages 4–8. Cells were all vimentin positive and were less than 15% positive for filamentous α-SMA (data not shown), consistent with a predominant fibroblastic phenotype with minimal smooth muscle (or myofibroblast) contamination.

### Mechanical stimulation of cultured fibroblasts

Cells were cultured in type I collagen precoated, six-well BioFlex silastic bottomed culture plates, seeded at a density of 10^5^ cells/well in DMEM/FBS. After 24h in static culture, the medium was changed and the cells were subjected to mechanical strain using a Flexercell-4000T TensionPlus (Flexcell International, Mckeesport, PA, USA). A sinusoidal cyclical strain of 30% amplitude was applied at a frequency of 12 cycles per minute for 24, 48 or 96h. The culture medium was not changed during the experiment and cells and conditioned media were harvested at each time point. The system was used without loading posts to enable microscopic evaluation post-stretching and as a consequence the strain experienced by the cells is a gradient of biaxial strain from 0% at the centre of the well, increasing towards a maximum at the periphery [15]. Strain at the maximal amplitude (30%) corresponds approximately to a deep inspiration to total lung capacity [16]. Control cells were cultured in the Bioflex plates under the same conditions but without mechanical strain.

### Extraction and purification of total RNA and mRNA quantification using RT-qPCR

Total RNA was extracted using the Trizol reagent (Invitrogen, Paisley, UK) according to the manufacturer’s protocol and samples were treated with RNase-free DNase (Ambion, Huntingdon, UK). The quality of RNA was determined using a NanoDrop spectrophotometer (Thermo Fisher Scientific, Loughborough, UK). 1 μg of total RNA was reverse-transcribed to cDNA using random hexamers and oligo (dT) primers and Moloney murine leukaemia virus (MMLV) reverse transcriptase enzyme (PrimerDesign, Southampton, UK) according to the manufacturer’s instructions.

Quantification of cDNA was performed by real-time polymerase chain reaction (PCR) using an iCycler IQ™ (Bio-Rad, Hertfordshire, UK). Gene specific primers for collagens I (COL1A1) and III (COL3A1), versican, biglycan, decorin, IL-8 and αSMA were used in conjunction with SYBR Green or as a fluorogenic ‘PerfectProbe’ (PrimerDesign, Southampton, UK); for those assays employing SYBR Green, melt curve analysis was also performed. Data were normalized to GAPDH and expressed relative to the median value of the non-strain non-asthmatic group using the ΔΔCT method.

### Cell number determination

Viable cell counts were established using trypan blue exclusion and evaluation by direct cell counting using a haemocytometer.

### IL-8 enzyme-linked immunoassay

IL-8 released into cell culture supernatants was measured by enzyme-linked immunosorbent assays (ELISA) according to the manufacturer’s protocols (IL-8, R&D, Abingdon, UK).

### Soluble collagen assay

Total collagen secreted from cultured cells was determined using the Sircol soluble collagen assay (Biocolor, Belfast, Northern Ireland). In brief, 4M NaCl was added to 750μl of test sample to precipitate the collagen which was harvested by centrifugation at 12,000 g for 10 min. The pellets were re-solubilised in 0.5M acetic acid before mixing with 1ml of Sirius red dye, an anionic dye that reacts specifically with basic side-chain groups of collagens, for 30 minutes. The precipitated collagen-dye complex was harvested by centrifugation at 12,000 g for 10 minutes, and the bound dye released using alkali reagent before measurement of its absorbance at 540 nm. Collagen was quantified relative to a standard curve (0–12.5 μg collagen; limit of detection = 2.5 μg).

### Zymography

The release of MMP2 and MMP9 into the media was detected using gelatine zymography. Culture supernatants from fibroblasts treated with TGFβ2 were used as a positive control. Samples were loaded on to 10% w/v polyacrylamide gels containing 1mg/mL gelatine with non-reducing Laemmli’s buffer. After electrophoresis, gels were renatured with 2.5% triton-X, before rinsing and incubating overnight in developing buffer at 37°C. Gels were stained with Coomaassie Brilliant Blue and de-stained prior to analysis using ImageJ (NIH, Bethesda, MD).

### SDS-PAGE and Western blotting

After exposure to mechanical strain for 96h, cell pellets were lysed in SDS buffer plus protease inhibitor cocktail. The lysates were harvested, sonicated and samples (20 μg protein) in Laemmli’s sample buffer plus 5% 2-mercaptoethanol were heat denatured prior to electrophoresis using SDS polyacrylamide gels. Separated proteins were transferred onto Hybond-ECL membranes (Amersham, Buckinghamshire, UK). Primary antibody incubations were performed overnight at 4°C with anti-α-SMA antibody or anti-GAPDH antibody (both supplied by Sigma, Poole UK). Membranes were probed with horseradish peroxide conjugated secondary antibodies (Dako, Cambridge, UK), with detection via enhanced chemiluminescence (ECL) (Amersham, Buckinghamshire, UK) according to the manufacturer’s instructions. All blots were quantified using ImageJ (NIH, Bethesda, MD, USA).

### Fluorescent staining for F-actin and α smooth muscle actin

Samples were fixed with 4% paraformaldehyde in PBS for 15 minutes at room temperature, permeabilized in 0.1% Triton in PBS for 5 min and then blocked with PBS containing 1% BSA and 0.1% Triton-X 100. F-actin was visualized by staining using Alexa Fluor488® conjugated Phallodin (1:150) (Invitrogen, Paisley, UK). α smooth muscle actin (αSMA) immunostaining was performed with a FITC conjugated mouse monoclonal anti- αSMA antibody (1:1000) (SIGMA, Poole, UK). Cells were examined using a Leica epifluorescent microscope (Leica, Milton Keynes, UK).

### Statistical analysis

The data were analysed using GraphPad Prism 6.0 (GraphPad Software Inc). Normal distribution of data was evaluated using the Shapiro Wilk test; parametric data are presented as mean± standard deviation while non-parametric data are displayed as median and interquartile range. Paired data from strained and non-strained cells were analysed by paired Student’s t test or Wilcoxon signed rank test for parametric or non-parametric data respectively. For the analysis of unpaired groups, a Two Sample t-test or Mann-Whitney U test was employed for parametric and non-parametric data respectively. Results were considered statistically significant at p < 0.05. All data is available online, see S1 Data.

## Results

### Characterization of the effects of mechanical strain on fibroblast morphology and proliferation

**Fig 1:**
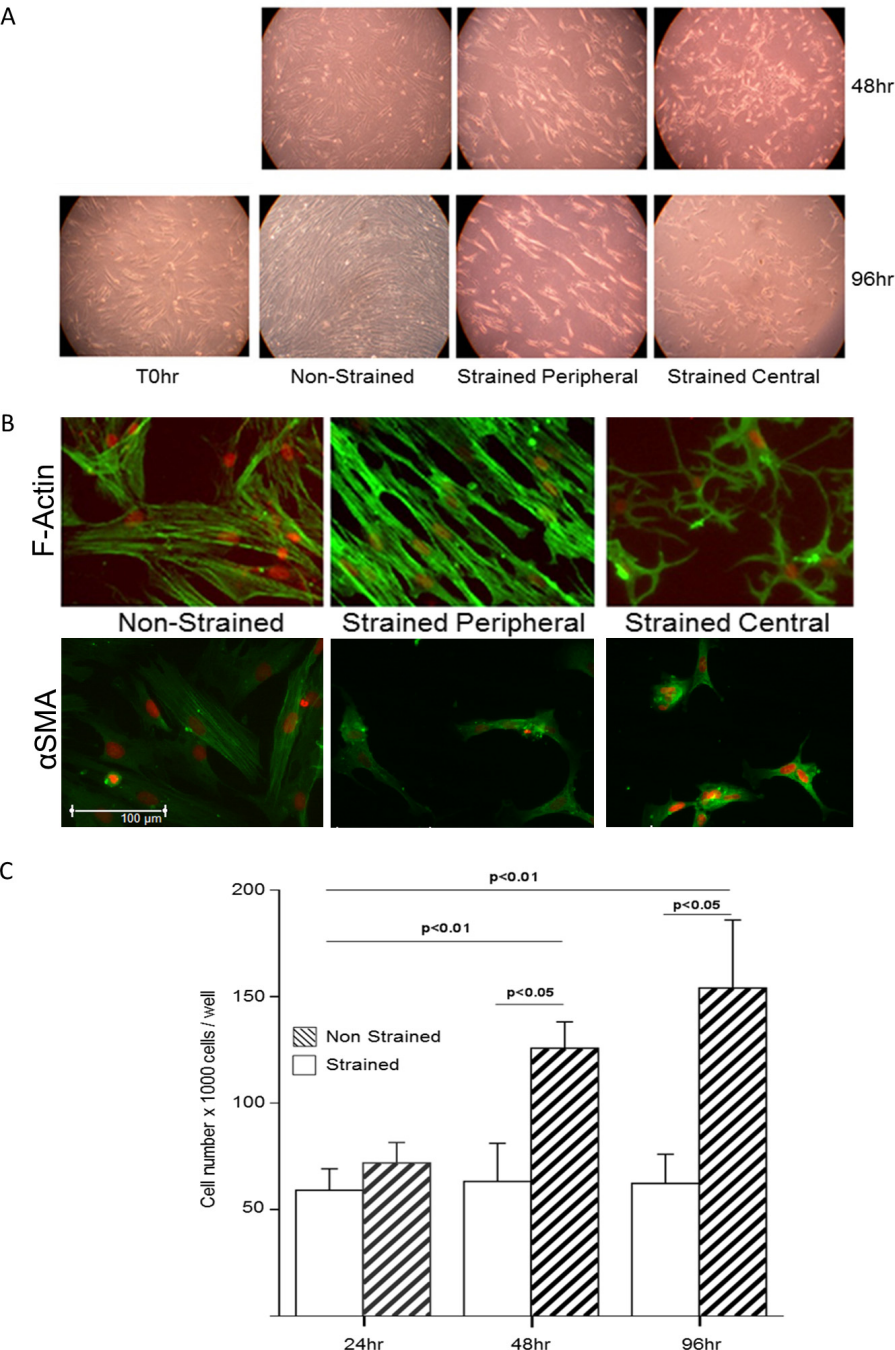
The effect of cyclical mechanical strain on bronchial fibroblast morphology and cell number. A) Photomicrographs showing fibroblast morphology using phase contrast microscopy. For the strained cells, two views are shown demonstrating the differences in morphology in the centre or periphery of the culture well. B) Localization of F-actin stress fibres using FITC-conjugated phalloidin (green) and α smooth muscle actin (αSMA) using a FITC-conjugated mouse monoclonal anti αSMA antibody with 7AAD nuclear staining (red). Scale bar = 100μm. C) Cell numbers (mean+SD) for three individual fibroblast cultures (1 normal and 2 asthmatic donors). Cell number was determined by direct cell counting at 24, 48 or 96h. The difference between strained and non-strained cell numbers was tested for statistical significance using a paired-t test, and the differences between time points was tested for statistical significance using a one way ANOVA.

Throughout this study, fibroblasts were exposed to cyclical equi-axial mechanical strain (30% and 12 cpm) for 24-96h. Initial experiments were performed to characterize the effects of mechanical strain on cell morphology and number (Fig 1). The effect of mechanical strain was dependent on the position of the cells within the well, reflecting the degree of strain they experienced; those at the periphery (high strain) had an elongated morphology and were aligned parallel to each other whereas those in the middle of the well (minimal strain) tended to be more stellate (Fig 1A). In contrast, non-strained control cells had a random orientation. These observations were supported by analysis of the F-actin stress fibres or αSMA in the cells (Fig 1B). In all of these studies, no differences were observed in the behaviour or phenotype of the fibroblasts from non-asthmatic or asthmatic donors. The cell number was determined by direct cell counting. Consistent with their morphological appearance, non-strained fibroblasts showed a significant increase in cell number over 96h; in contrast, there was no significant change in cell number in the strained cells over the duration of the experiment (Fig 1C).

### Mechanical strain enhances the production of collagen but not proteoglycan expression

**Fig 2:**
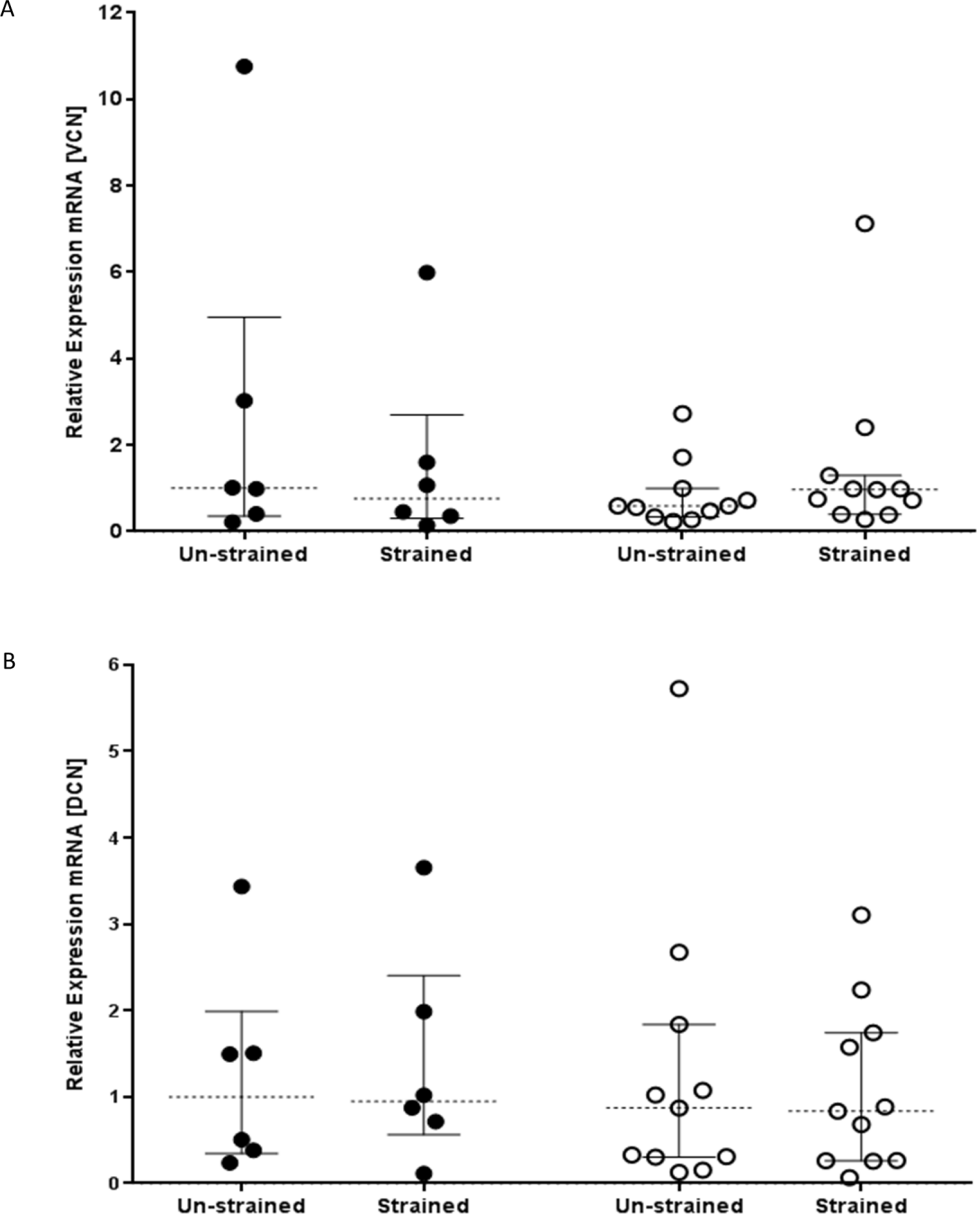
Comparison of the effect of cyclical mechanical strain on fibroblast proteoglycan expression. Fibroblasts derived from bronchial biopsies from non-asthmatic (n = 6, filled circles) or asthmatic donors (n = 11, open circles) were exposed to cyclical mechanical strain for 48h. RNA was extracted and RT-qPCR performed to measure expression of A) versican (VCN) and B) decorin (DCN). Data were normalized to GAPDH and displayed relative to the median of the non-asthmatic non-strain group using the ΔΔCT method. The data were analysed using Wilcoxon’s signed rank test. No differences were found between the treatments or subject groups.

To determine the effect of mechanical strain on ECM production, we measured proteoglycan and collagen I and III mRNA expression. In pilot studies, examining gene expression over 6-144h, mechanical strain caused an increase in collagen mRNA expression at 48h and 96h compared with unstrained cells, however no change in proteoglycan expression was noted at any time point (data not shown). Consequently, for the study, we chose to compare the response of fibroblasts from asthmatic and non-asthmatic donors at 48 and 96hr, with mRNA results displayed for samples harvested at 48h and protein data at 96h. As observed in the pilot study, there was no significant change in versican (VCN) and decorin (DCN) mRNA expression (Fig 2A and 2B respectively) after mechanical strain in either the non-asthmatic or asthmatic group.

**Fig 3:**
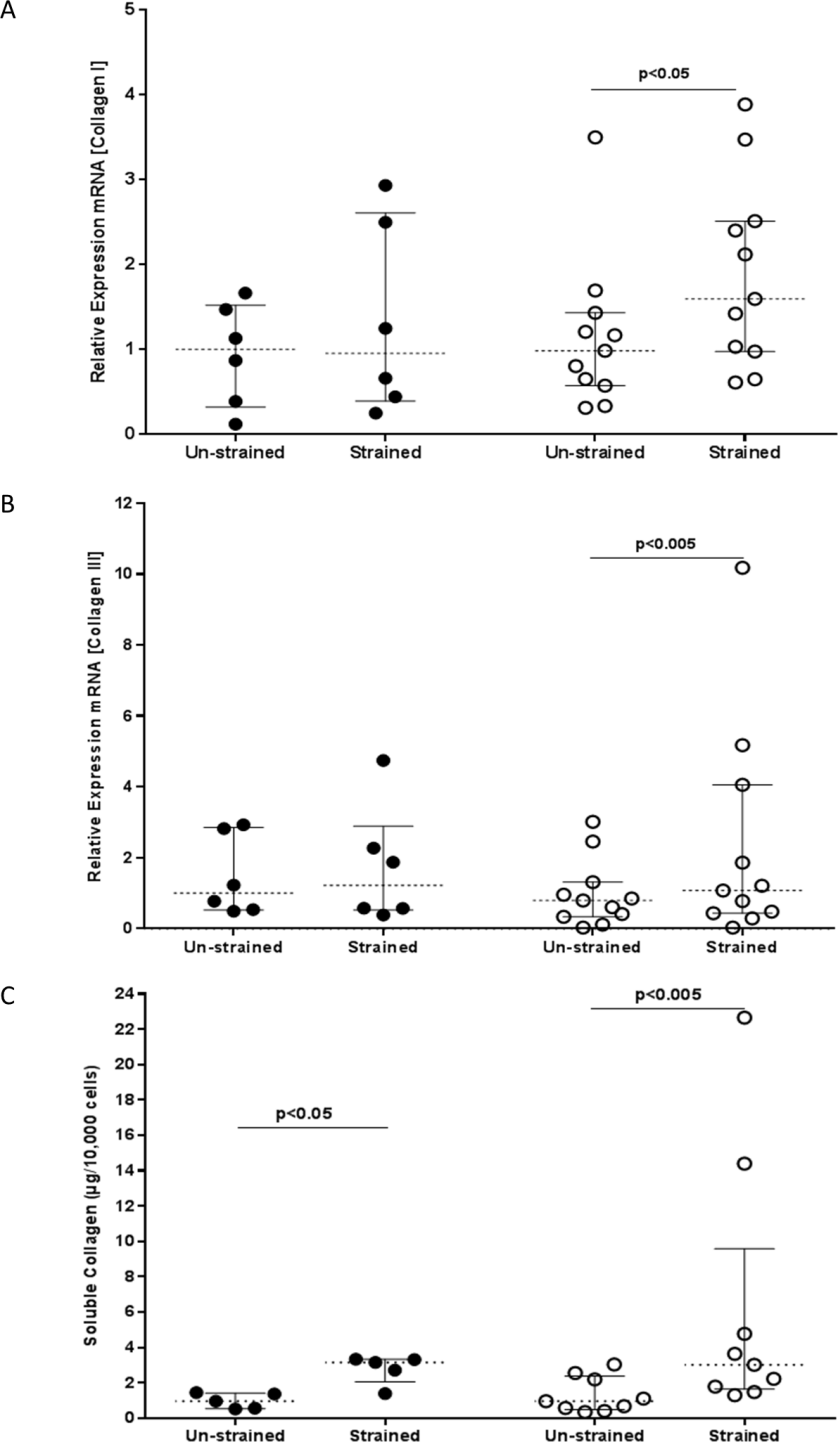
Mechanical strain enhances the production of interstitial collagens. Expression of A) collagen I (COL1A1) and B) collagen III (COL3A1) mRNA was determined using RT-qPCR after exposing fibroblasts from non-asthmatic (filled circles) or asthmatic (open circles) donors to cyclic strain or no strain for 48h. Data were normalized to GAPDH and expressed relative to the median value of the non-strain non-asthmatic group using the ΔΔCT method. The expression of collagen in cell culture supernatants C) was measured at 96h using the Sircol dye binding method. The data were analysed using Wilcoxon’s signed rank test. The results for the RT-qPCR represent data for bronchial fibroblasts from non-asthmatic (n = 6) or asthmatic (n = 11) subjects, and for the soluble collagen assay data for fibroblasts from non-asthmatic (n = 5) or asthmatic (n = 9) donors. The group sizes for the soluble collagen experiment were reduced to 5 for non-asthmatic and 9 for the asthmatic groups respectively, as a result of inadequate samples to perform analysis.

A comparison of collagen I and III mRNA expression after 48h of mechanical strain showed an increases in both collagen I (p<0.05) and collagen III (p<0.005) (Fig 3A and 3B respectively) only in fibroblasts from asthmatic donors. The increase in mRNA expression was confirmed at the protein level (96h) with cells from asthmatic donors showing a significant increase (p<0.005) in the level of soluble collagen in culture supernatants after exposure to mechanical strain (Fig 3C). Even though no change in collagen mRNA expression was observed at 48h in cultures from non-asthmatic donors, there was an increase in soluble collagen (p<0.05) detectable at 96h in response to strain in this group.

### Mechanical strain suppresses αSMA expression

**Fig 4:**
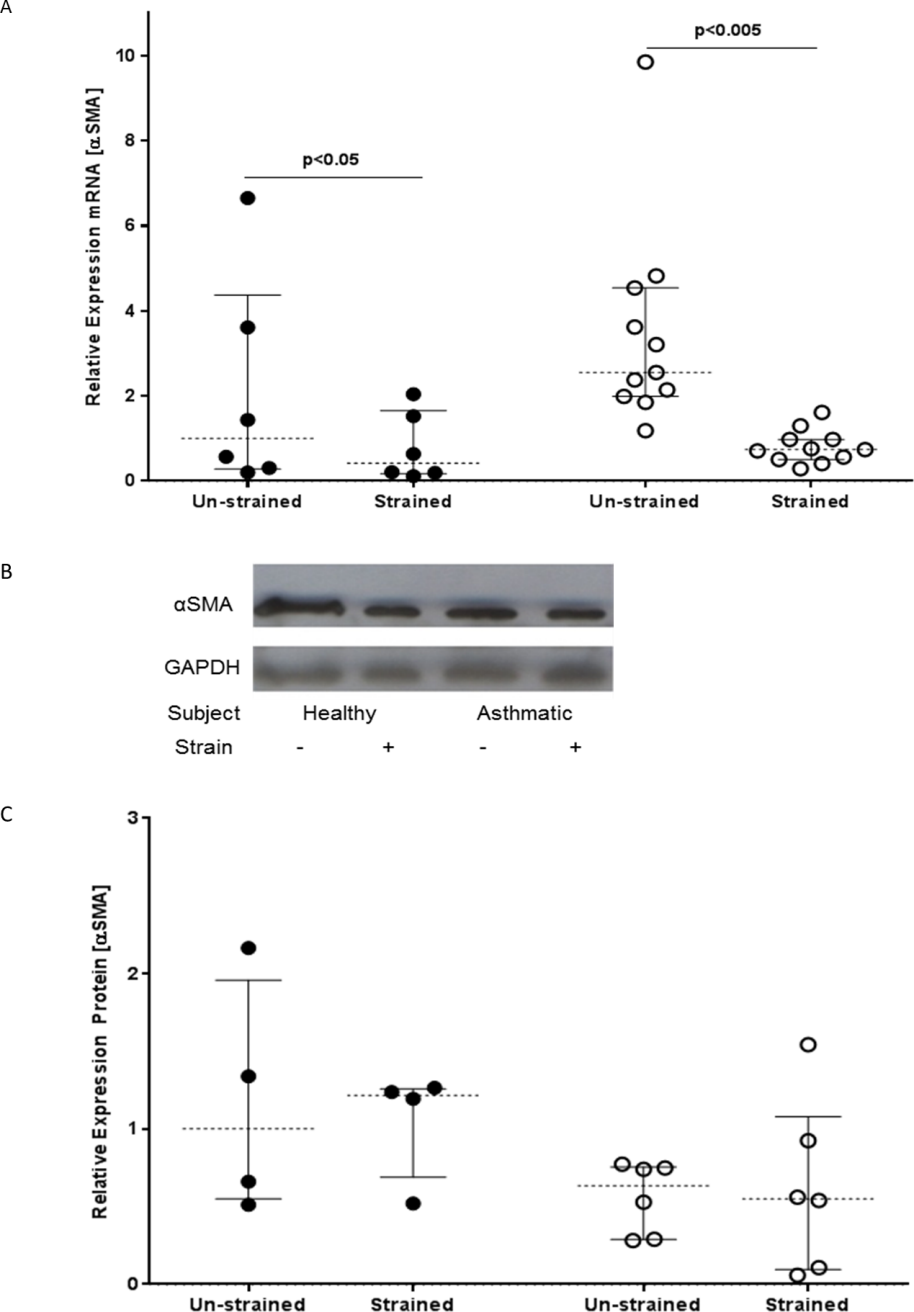
The effect of mechanical strain on αSMA mRNA and protein expression as a marker of myofibroblast phenotypic change. Fibroblasts from non-asthmatic (filled circles) or asthmatic (open circles) donors were exposed to cyclical mechanical strain or left untreated for 48 or 96h. A) Expression of αSMA mRNA was measured by RT-qPCR (48h); data were normalized to GAPDH and displayed relative to the median of the unstrained non-asthmatic group using the ΔΔCT method. B) αSMA protein expression was assessed by Western blotting (96h); the figure shows a representative blot using fibroblasts from one asthmatic and one non-asthmatic donor cultured in the absence or presence of strain. C) αSMA protein expression was determined by semi-quantitative analysis using densitometry with normalisation relative to GAPDH protein expression. The data were analysed using Wilcoxon’s signed rank test. The results shown for the qRT-PCR represent data for fibroblasts from non-asthmatic (n = 6) or asthmatic (n = 11) donors and for the Western blotting for fibroblasts from non-asthmatic (n = 4) or asthmatic (n = 6) donors.

Since we found an increase in expression of interstitial collagens after mechanical strain, we assessed whether strain also triggered myofibroblast differentiation by measuring αSMA mRNA expression at 48h. Contrary to expectations, this revealed that strain caused a significant suppression of αSMA mRNA expression in fibroblasts from asthmatic (p<0.005) or non-asthmatic subjects (p<0.05) (Fig 4A). Analysis of αSMA protein (96h) using Western blot analysis indicated that there was no significant difference in αSMA protein expression between the strained and un-strained cells (Fig 4B and 4C), suggesting no overall change in phenotype.

### Mechanical strain induces IL-8 mRNA and Protein Expression

**Fig 5:**
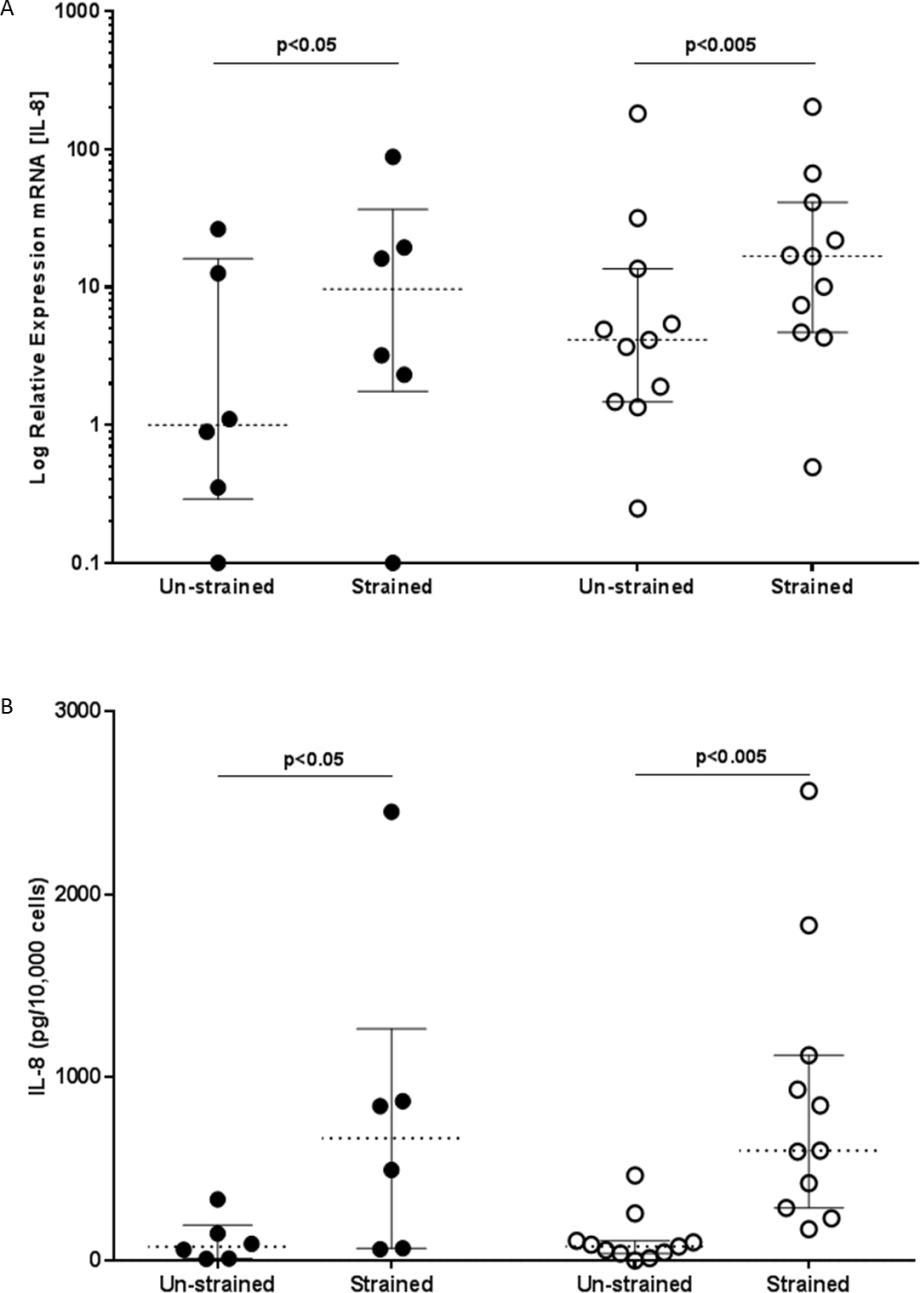
Mechanical strain induces IL-8 expression. Fibroblasts from non-asthmatic (filled circles) or asthmatic (open circles) donors were cultured in the absence or presence of cyclical mechanical strain for 48 and 96h. A) IL-8 mRNA expression at 48h was assessed by RT-qPCR with the data normalized to GAPDH and displayed relative to the median of the unstrained non-asthmatic group using the ΔΔCT method. B) Release of IL-8 protein into the cell culture supernatants was assessed after 96 h by ELISA and normalised for cell number. The data were analysed using Wilcoxon’s signed rank test. Results shown represent data for fibroblasts from non-asthmatic (n = 6) or asthmatic (n = 11) donors for both the RT-qPCR and ELISA.

Airway inflammation and remodelling are both believed to play an important role in asthma pathogenesis. To investigate whether mechanical strain also induced a proinflammatory response, we measured IL-8 mRNA and protein expression by RT-qPCR (Fig 5A) and ELISA (Fig 5B). In both non-asthmatic and asthmatic groups, fibroblasts exposed to mechanical strain demonstrated significant increases in IL-8 mRNA (p<0.05 and p<0.005 respectively) at 48h and protein release at 96h (p<0.05 and p<0.005 respectively) compared with unstrained cells. There was no significant difference when comparing the mechanically strained fibroblasts from asthmatic and non-asthmatic donors for IL-8 mRNA or protein.

### Mechanical strain induces protease expression

**Fig 6:**
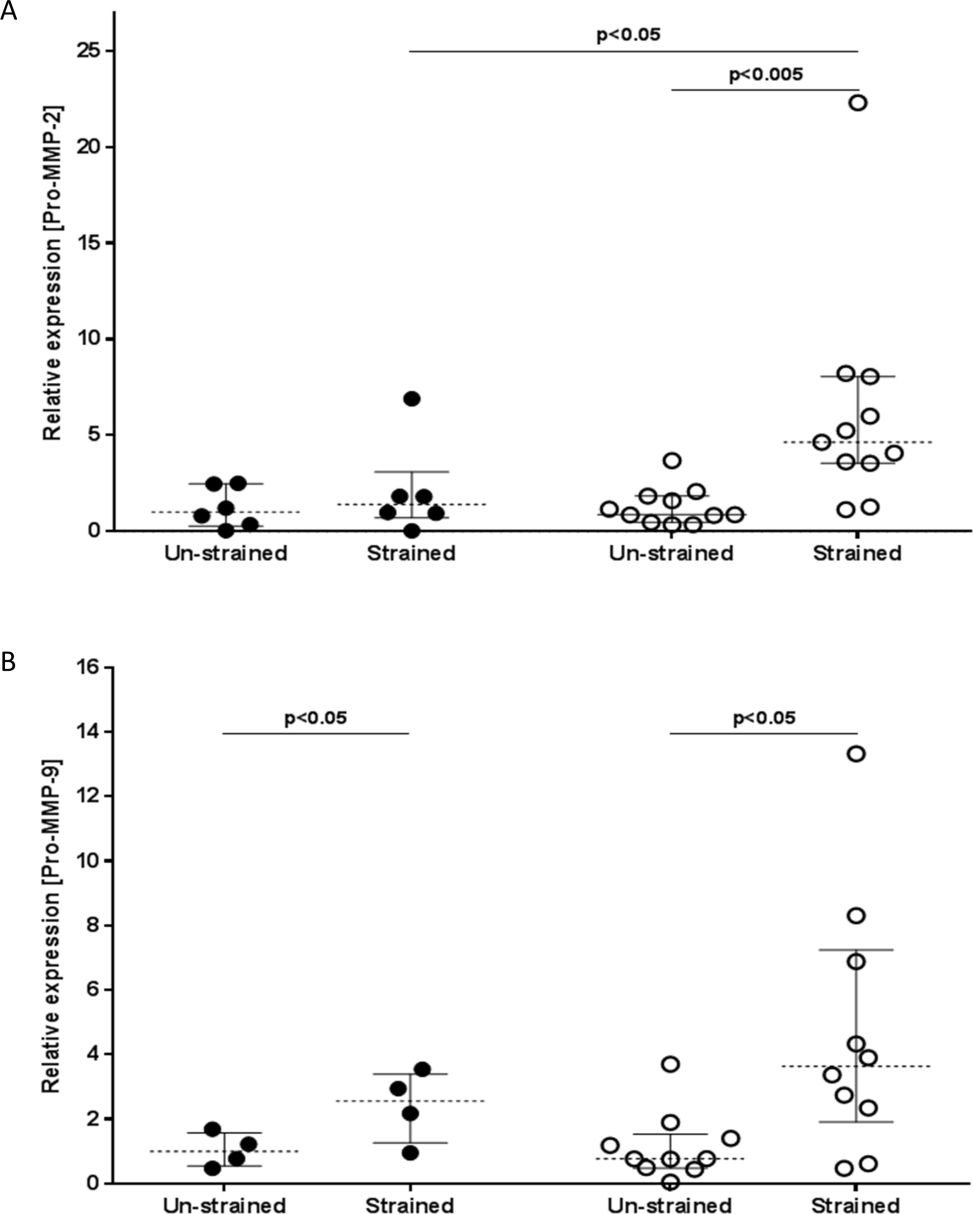
Mechanical strain induces protease expression. Fibroblasts derived from non-asthmatic (filled circles) or asthmatic (open circles) donors were subjected to mechanical strain (or left unstrained) for 96h. The resultant culture supernatants were analysed for A) the 72-kDa pro-enzyme form of MMP-2 (ProMMP-2) and B) the 92 kDa pro-enzyme MMP-9 (ProMMP-9) using gelatine zymography and densitometry. Data were normalized per 10,000 cells and displayed relative to the median of the unstrained non-asthmatic group. The data were analysed using Wilcoxon’s signed rank test. Results shown are from fibroblasts from non-asthmatic (n = 6) or asthmatic (n = 11) donors for ProMMP-2, and fibroblasts from non-asthmatic (n = 4) or asthmatic (n = 10) donors for ProMMP-9.

ECM degradation by matrix metalloproteinases (MMPs) is important for tissue remodelling. To determine the effect of mechanical strain on MMP expression, culture supernatants were analysed using gelatine zymography (Fig 6A and 6B respectively). Results showed a small amount of active MMP-9, the level of which were unaffected by mechanical strain; however there was a significant increase in proMMP-9 in culture supernatants of fibroblasts from either asthmatic or non-asthmatic donors (P<0.05 and P<0.05 respectively) following mechanical strain. For MMP-2, only the fibroblasts from asthmatic donors showed a significant increase in proMMP-2 following mechanical strain P<0.005 and no active MMP-2 was detected. As a control, TGFβ treated fibroblasts showed a large increase in active MMP-2 (See S1 Fig)). As fibroblasts produced MMP2 and MMP9, we excluded the possibility that the observed increase in soluble collagen was not an indirect effect of MMPs degrading exogenous collagen from the BioFlex silastic bottomed culture plates. Thus, incubation of conditioned medium from strained cells in new wells coated with type I collagen or BSA revealed no significant difference in levels of soluble collagen in the conditioned medium, consistent with the predominant expression of inactive pro-MMP2.

## Discussion

Creation of mechanical tension within the lungs is critical for inspiration and expiration and is thought to provide regulatory signals that govern the composition of the pulmonary vasculature and airways [17, 18]. Furthermore, it has been observed that deep inspiration has a bronchoprotective effect which can prevent bronconstriction [19]. While the ECM plays an important role in determining the normal mechanical properties and elasticity of a tissue, abnormal ECM remodelling with deposition of interstitial collagens in the *lamina reticularis* and submucosa of the airway wall is one of the hallmarks of asthma. Since abnormal mechanical loads associated with compression can affect diverse cellular functions including cell proliferation and alteration of the composition of the ECM leading to fibrosis [6], we postulated that the same may also be true for mechanical strain. In addition we postulated that there would be a difference in responses of airway fibroblasts from asthmatic and non-asthmatic donors. Thus, we used primary airway fibroblasts from asthmatic and non-asthmatic donors and exposed them to mechanical strain at a maximal amplitude corresponding approximately to deep inspiration to total lung capacity [16]. Although it should be noted that deep inspirations are infrequent and tidal breathing applies much lower strains, increased ventilation is seen in endurance sports men/women especially elite swimmers and heavy exercise has been associated with asthma-like symptoms (34). Our results indicate that cyclical mechanical strain enhances the profibrotic and inflammatory responses of airway fibroblasts, consistent with a potential to exacerbate airway inflammation and remodelling in asthma.

We have demonstrated that cyclical mechanical strain of airway fibroblasts enhances profibrotic responses by increasing the production of interstitial collagens, a finding consistent with several other studies that have reported that mechanical strain increases collagen expression [8, 20]. However, we failed to detect any changes in mRNA expression of the proteoglycans, versican and decorin which does contrast with previous reports that mechanical strain to airway fibroblasts from either non-asthmatic or asthmatic donors promoted proteoglycan mRNA expression [10]. However, in these earlier experiments the analysis was semi-quantitative and depended on densitometry of PCR products following gel electrophoresis.

While no changes were demonstrated in the expression of proteoglycans, significant increases in interstitial collagens I and III mRNA following strain were demonstrated for the asthmatic group. While increases in mRNA expression were not observed in the non-asthmatic group, significant increases were apparent at the protein level in both groups, as determined by soluble collagen measurement. Failure to see a change in mRNA expression in the non-asthmatic group may have been due to the smaller group size, the time point selected for analysis of mRNA expression, or to engagement of post-transcriptional regulatory mechanisms [21]. There is considerable evidence to support the induction of interstitial collagen particularly type 1 collagen by mechanical strain [6–8]. Although Blaauboer *et al* found a decrease in collagen mRNA expression after exposure of primary lung fibroblasts to mechanical strain, they used less strain (10% and 6 cycles per minute) [22] suggesting that the amount of mechanical stimulation may also have an impact on fibroblast responses. This implies that an increase in ECM production may occur as a pro-fibrogenic response only after exposure of airway fibroblasts to higher, albeit still physiological [16], levels of mechanical stimulation. Our data demonstrate that fibroblasts from both non-asthmatic and asthmatic individuals responded similarly to strain in terms of collagen protein production.

We postulated that the increased level of collagen expression could be attributed to myofibroblast differentiation, but we found that mechanical strain of fibroblasts in monoculture did not enhance αSMA expression, a marker of myofibroblast differentiation. These findings are consistent with a previous report that mechanical strain suppresses αSMA expression in pulmonary fibroblasts [22] but contrast with a previous study using an epithelial and fibroblast co-culture model [20]. The use of a monoculture rather than a co-culture model may affect the expression of αSMA, as epithelial cells are a significant source of TGF-β after injury/stimulation [23]. This epithelial-derived TGF-β could overcome the suppressive effect of mechanical strain on myofibroblast differentiation which we observed in the monoculture. Although we were able to detect small quantities of active TGF-β1 in a limited number of fibroblast cultures undergoing strain (data not shown), this was not a universal response and is consistent with limited evidence of myofibroblast differentiation. An alternative explanation for the observed reduction αSMA may be via the down regulation of Endothelin-1 (ET-1) independent TGF-β1 [24].

It is also possible that different modes of supplied strain can result in differing effects. In our study, we focused on the role of radial cyclic strain to mimic forces associated with deep inhalation while the study Choe *et al*., 2006 was concerned with modelling the effects of compressive force associated with bronchoconstriction [20]. It has also been reported that uniaxial stretch can cause murine embryonic mesenchymal cells to undergo smooth muscle differentiation which is also characterized by an increase in αSMA expression [25]. While this difference might, in part, be explained by differences in plasticity between adult and embryonic fibroblasts, αSMA is known to be a mechano-sensitive protein that is recruited to stress fibres under high tension. It is possible that high levels of cyclic strain repetitively disrupt and/or disorganise the cytoskeleton and cell matrix adhesions [26].

It has been proposed that mechanical forces can activate fibroblasts to induce a pro-fibrogenic phenotype with an increase in proliferation, ECM synthesis, and protease activities [27]. However other studies have shown that fibroblast proliferation is unaffected and is dependent on either the frequency or strain profile [28]. We found that fibroblasts failed to proliferate when exposed to 30% biaxial strain while they proliferated normally in static culture. Nishimura *et al*. have also reported that mechanical strain caused a decrease in cell number due to suppression of DNA synthesis with no effect on apoptosis [29]. Indeed consistent with these previous observations, we saw a reduction in proliferation and no observable apoptosis. While the cells remodel their cytoskeleton in response to strain the overall cytoskeletal proteins may remain constant. Reorganization of cytoskeleton within the cell may require protein synthesis; this would be considerably less than the synthesis requirements of dividing cells.

While myofibroblast differentiation was not demonstrated in the present work, there was a clear increase in the expression of both collagen mRNA and protein. We believe that the increase in collagen may reflect increased biosynthetic activity of non-proliferative fibroblasts *per se* rather than myofibroblast transformation. It has been reported that collagen synthesis is highest in low density cell cultures [30], suggesting that there may be a link between decreased proliferation and increased biosynthetic capacity. We also found that mechanical strain altered fibroblast morphology but this effect was highly dependent on the location of the cells in the culture well and probably reflects the extent to which the cells experience strain. In previous studies, cells grown at 6–10 mm from the centre of the well were found to experience the maximum strain, whereas cells grown at 0–5 mm from the centre of the well experienced little to no strain [15, 31]. It has also been shown that fibroblasts sense mechanical strain via integrin adhesion receptors which connect the cytoskeleton stress fibres to the ECM [32]. Consistent with this, we found that mechanical strain caused rearrangement in F-actin stress fibres. This is further supported by a recent study suggesting that any alteration in fibroblast morphology is directly related to the degree of actin polymerization [33].

In addition to the pro-fibrotic influence of mechanical strain, the presented work also demonstrated an increase in production of the neutrophil chemotactic factor, IL-8, by airway fibroblasts after exposure to cyclical mechanical strain. Neutrophilic airway inflammation is seen most commonly in patients with severe refractory asthma [34] and has been reported in autopsies of patients who died soon after the onset of a severe exacerbation [35]. Airway neutrophilia has also been demonstrated in exercise-induced bronchoconstriction [36], and in healthy non-asthmatic endurance athletes such as marathon runners and long distance swimmers, both at baseline and following heavy exercise [37]. The usual respiratory response to exercise is an increase in tidal volume at low-to-moderate workloads, or an increase in respiratory frequency at high levels of exercise [36]. This may result in increased mechanical stimulation to the airways causing more IL-8 to be produced, leading to more neutrophil infiltration into the airways. Consequently, the ability of mechanical strain to stimulate airway fibroblast IL-8 production may contribute to the neutrophilic airway inflammation in athletes, severe fatal asthma, and exercise induced asthma.

Mechanical strain also caused an induction in MMP expression in both groups of fibroblasts consistent with a change in matrix turnover. However, there was a divergence between the two groups, with pro-MMP 2 and 9 both being induced by strain in the asthma-derived fibroblasts, whereas only pro-MMP 9 was induced by strain in the non-asthmatic group. While the group sizes differed, there is no evidence that this is the source of the difference between the two groups. Of note, there was little induction of active MMPs by strain, even though TGFβ treatment was able to cause a marked upregulation of active MMP2 in the fibroblasts. MMP-2 and 9 overlap considerably in terms of function, both acting to degrade basement membrane collagen type IV and a number of other overlapping collagens and proteoglycans [38]. Both MMP-2 and MMP-9 can release matrikines, cytokine-like molecules resulting from the degradation of ECM components such as elastin and collagen [39]; there is considerable redundancy in the regulation of MMP- 2 and 9. However, the overlap is not complete. MMP-9 has been linked with the activation of several cytokines such as TGFβ, IL1β and is also suggested to be involved in the action of IL-8 on neutrophil signalling. In contrast, MMP-2 has been suggested to cleave immature collagen 1, CCL7 (c-c motif ligand; previously known as monocyte chemoattractant protein-3, MCP3) leading to a reduction in inflammation [40]. This suggests that there may be a functional consequence in the different levels of expression of MMP2 seen between the two groups. We suggest that there is a mutual alteration or change in matrix turnover and that MMP-2 and 9 may have opposing functionality in regard to inflammation, with MMP-9 being pro-inflammatory and MMP-2 being anti-inflammatory in terms of cellular recruitment to the lung [41].

In the current study, we have demonstrated that mechanical strain leads to significant induction of matrix proteins in the form of collagen I and III as well as increases in pro-MMPs 2 and 9 and inflammatory cytokine IL-8, at the mRNA and protein level. As there were no substantial differences in the responses of fibroblasts from asthmatic or non-asthmatic donors, our data suggest that these are normal responses to mechanical strain. However there were some modest differences in the regulation of the interstitial collagens I / III and MMPs between the groups suggesting some form of genetic or epigenetic regulation. These difference may contribute to remodelling, for example in patients with chronic cough who have been shown to have a thicker subepithelial basement membrane than non-asthmatic control subjects [42]. In a recent *in vivo* challenge study in asthma, it has been shown that there is an increase in subepithelial collagen in asthmatic airways following methacholine-induced bronchoconstriction consistent with a role for mechanical forces in driving remodelling responses [4]. As there are significantly more fibroblasts in the *lamina reticularis* of the asthmatics subjects [43], we suggest that the cumulative effects of the increased fibroblast numbers in combination with mechanical strain may make this mode of matrix production, turnover and inflammatory regulation an important consideration in asthma.

## Supporting Information

S1 Data Primary data from manuscript Figs 1–6 and patient clinical data. (XLSX)

S1 Fig Representative Zymogram of Increased MMP Expression in Response to Mechanical Strain. (TIF)
