# Supplementary figures and images for: Mechanical Strain Causes Adaptive Change in Bronchial Fibroblasts Enhancing Profibrotic and Inflammatory Responses
Source: PLoS One. 2016 Apr 21;11(4):e0153926. doi: 10.1371/journal.pone.0153926 (PMC4839664; doi:10.1371/journal.pone.0153926)

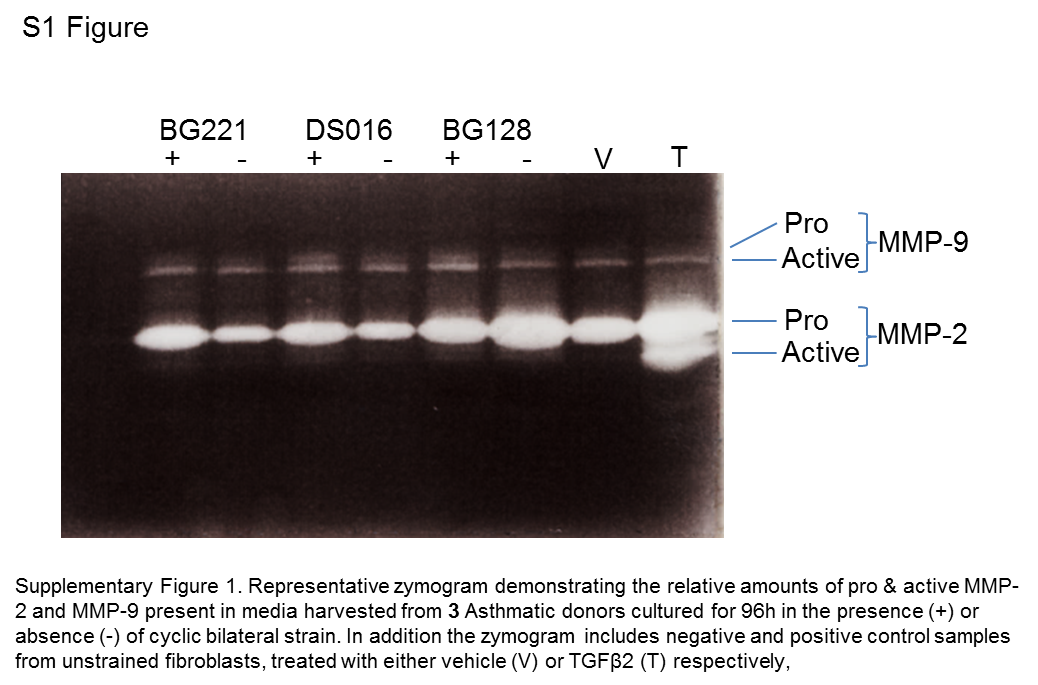

Supplement: S1 Fig — (TIF) [file pone.0153926.s002.tif]
